# Basal forebrain-lateral habenula inputs and control of impulsive behavior

**DOI:** 10.1038/s41386-024-01963-7

**Published:** 2024-08-18

**Authors:** Eun-Kyung Hwang, Agustin Zapata, Vivian Hu, Alexander F. Hoffman, Hui-Ling Wang, Bing Liu, Marisela Morales, Carl R. Lupica

**Affiliations:** 1 Computational and Systems Neuroscience Branch, Electrophysiology Research Section, U.S. Department of Health and Human Services, National Institutes of Health, National Institute on Drug Abuse Intramural Research Program, Baltimore, MD 21224 USA; 2 Integrative Neuroscience Research Branch, Neuronal Networks Section, U.S. Department of Health and Human Services, National Institutes of Health, National Institute on Drug Abuse Intramural Research Program, Baltimore, MD 21224 USA; 3 Present Address: Department of Behavioral Neuroscience, Oregon Health Sciences University, Portland, OR 97239 USA

**Keywords:** Neurotransmitters, Synaptic transmission

## Abstract

Deficits in impulse control are observed in several neurocognitive disorders, including attention deficit hyperactivity (ADHD), substance use disorders (SUDs), and those following traumatic brain injury (TBI). Understanding brain circuits and mechanisms contributing to impulsive behavior may aid in identifying therapeutic interventions. We previously reported that intact lateral habenula (LHb) function is necessary to limit impulsivity defined by impaired response inhibition in rats. Here, we examine the involvement of a synaptic input to the LHb on response inhibition using cellular, circuit, and behavioral approaches. Retrograde fluorogold tracing identified basal forebrain (BF) inputs to LHb, primarily arising from ventral pallidum and nucleus accumbens shell (VP/NAcs). Glutamic acid decarboxylase and cannabinoid CB1 receptor (CB1R) mRNAs colocalized with fluorogold, suggesting a cannabinoid modulated GABAergic pathway. Optogenetic activation of these axons strongly inhibited LHb neuron action potentials and GABA release was tonically suppressed by an endogenous cannabinoid in vitro. Behavioral experiments showed that response inhibition during signaled reward omission was impaired when VP/NAcs inputs to LHb were optogenetically stimulated, whereas inhibition of this pathway did not alter LHb control of impulsivity. Systemic injection with the psychotropic phytocannabinoid, Δ^9^-tetrahydrocannabinol (Δ^9^-THC), also increased impulsivity in male, and not female rats, and this was blocked by LHb CB1R antagonism. However, as optogenetic VP/NAcs pathway inhibition did not alter impulse control, we conclude that the pro-impulsive effects of Δ^9^-THC likely do not occur via inhibition of this afferent. These results identify an inhibitory LHb afferent that is controlled by CB1Rs that can regulate impulsive behavior.

## Introduction

Choosing appropriate behavioral strategies to maximize reward enhances survival, and the use of environmental stimuli to predict reward availability is critical to engaging these behaviors. Adaptive response strategies often use past experience to maximize reward and minimize energy expenditure. One simple strategy involves using cues to determine when reward probability is high to then energize reward seeking. Conversely, when reward availability is improbable, responding should be curtailed because it is unproductive and consumes metabolic resources. Withholding unproductive responses is also fundamental to the concept of “self-control”, and alternatively, the absence of response inhibition represents a loss of control that is often defined as impulsive behavior.

Impulsivity is a key diagnostic feature of impulse control disorders (ICDs) that is observed in mood disorders, SUDs, ADHD, borderline personality disorder, bipolar disorder, and TBI [1–9]. The broad presence of impulsivity in neuropsychiatric disorders indicates a better understanding of brain cellular and circuit mechanisms is required to aid development of impactful therapeutic interventions. Many brain regions contribute to impulsivity, with impairments in cortical areas exerting “top down” control over subcortical brain regions emphasized previously [10, 11]. However, subcortical structures, such as amygdala, subthalamic nucleus, nucleus accumbens and striatum, are also implicated [10–12], and we recently report that the lateral habenula (LHb) plays a role in suppressing impulsive behavior [13].

Our previous studies used two rodent models of impulsivity. A Go/NoGo model of stimulus-controlled response inhibition was used to show that suppression of LHb activity or blockade of LHb muscarinic acetylcholine receptors (mAChRs) disrupts withholding of responding when cocaine seeking is not rewarded [13, 14]. Similarly, in a 5-choice serial reaction time task (5CSRTT), LHb inhibition or systemic exposure to either cocaine or Δ^9^-THC increased impulsivity, as shown by an increase in premature responding for reward [15]. This increased impulsivity with Δ^9^-THC or cocaine was also prevented by LHb CB1R antagonism [15], suggesting engagement of a LHb endocannabinoid (eCB) system by these drugs.

These studies are generally consistent with the idea that inhibition of LHb neurons increases impulsivity, and that mAChRs and the eCB system are involved [13–15]. Although few intrinsic sources of inhibition are found in LHb [16], extrinsic inhibitory input likely plays a role in regulating LHb-dependent behavior, and CB1Rs are found on some of these pathways [17–19]. Presently, we characterize an inhibitory LHb projection from the BF, originating in rostral ventral pallidum and caudal nucleus accumbens shell, that expresses CB1Rs, is inhibited by eCBs, and controls response inhibition in an operant task.

## Methods and materials

### Animals

Wildtype male Long-Evans rats (postnatal day 42–50), purchased from Charles River Laboratories were used in most experiments. Male and female LE-Tg(GAD1-Cre)3Ottc (GADCre rats) [20] were obtained from the NIDA-IRP transgenic breeding facility. For electrophysiological studies, injections of viral constructs were performed at postnatal day 52–57, and in vitro experiments conducted 7–8 weeks later. Animals were housed 2–4 per cage in a temperature and humidity-controlled facility and, unless stated, had *ad libitum* access to food and water. Animals in electrophysiological experiments had standard lighting conditions (lights on 0600 h, off 1800h). Those in behavioral experiments were housed in a reverse 12 h light/dark cycle, and behavioral procedures occurred during reversed dark phase. All procedures were designed using the “Guide for the Care and Use of Laboratory Animals” [21], and approved by our local animal care and use committee. The NIDA-IRP animal facility is accredited by the international Association for Assessment and Accreditation of Laboratory Animal Care (AAALAC).

#### Surgery

Surgeries were performed before starting behavioral training. Rats were anesthetized with isoflurane (4%, in 1 L/min O_2_) and placed in a stereotactic apparatus (Kopf Instruments). The isoflurane concentration was lowered to 1–1.5% (in 0.2 L/min O_2_) to maintain anesthesia, and core temperature was set at 37 °C using a heating pad. Incisions were closed with absorbable sutures, and body temperature was maintained until recovery from anesthesia. Rats were injected with an anti-inflammatory (meloxicam, 1 mg/kg, s.c.), before being returned to their home cage, and post-operative health assessments were performed for 3 days. Once normal feeding behavior resumed and weight gain observed for one-week after recovery, rats were singled housed for the remainder of the behavioral experiments.

Detailed Anatomical, behavioral, and electrophysiological methods are shown in Supplemental Materials.

## Results

### The LHb receives GABAergic afferents from Ventral Pallidum (VP) and Nucleus accumbens shell (NAcs) regions that express CB1R mRNA

**Fig. 1:**
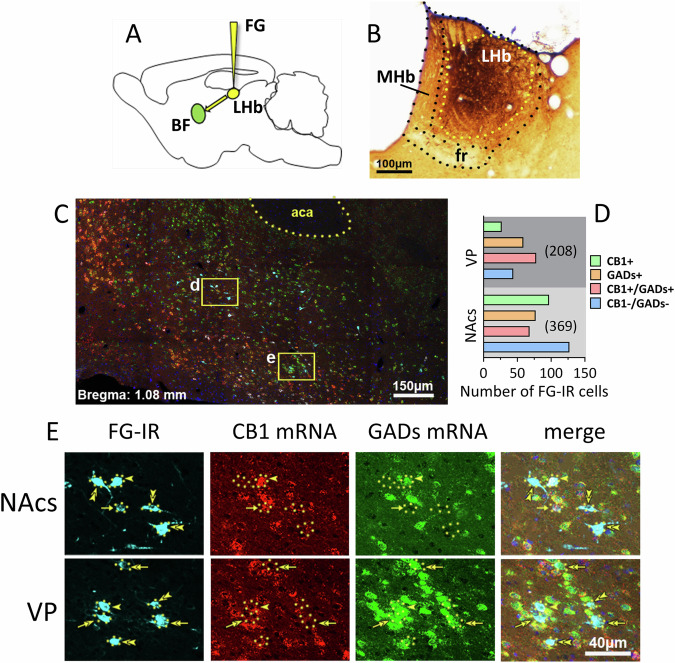
Basal forebrain (BF) neurons located in the nucleus accumbens shell (NAcs) and the ventral pallidum (VP) project to the LHb and express CB1R mRNA, GADs mRNA, or both. **A** Retrograde tracer FG was delivered into the LHb. **B** FG-IR in the injection site (brown). **C** Low magnification of a NAc shell (d) and VP (e) coronal section showing detection of FG-immunoreactivity (FG-IR, blue), expression of CB1 mRNA (red), and expression of GADs mRNA (green). These areas in yellow boxes are shown at higher magnification in the sequence shown in (**E**), top and bottom, respectively. **D** Number of FG-IR cells detected in VP and NAcs, sorted by co-expression of CB1R mRNA and/or GADs mRNA. A total of 208 FG-IR VP neurons projecting to the LHb were detected, and of these 24 (13%) expressed only CB1R mRNA, 59 (28%) expressed only GADs mRNA, 80 (38%) co-expressed CB1R and GADs mRNA, and 45 (21%) lacked both CB1R and GADs mRNA. A total of 369 FG-IR NAcs neurons projecting to the LHb were detected, and of these 97 (26%) expressed only CB1 mRNA, 79 (21%) expressed only GADs mRNA, 67 (18%) co-expressed CB1R and GADs mRNA, and 126 (34%) lacked both CB1R and GADs mRNA. FG-IR cell counts were made between +1.92 and +0.72 mm from bregma (*n* = 3 rats, 8–9 sections per rat). **E** NAcs and VP areas corresponding to boxes (**d**, **e**) in panel (**C**) at higher magnification. FG-IR neurons expressing CB1R mRNA without GADs mRNA are indicated by single arrows, FG-IR neurons expressing GADs mRNA and CB1R mRNA are indicated by single arrow heads, and double arrow heads indicate FG-IR without GADs or CB1R mRNA in NAcs. A FG-IR neuron expressing GADs mRNAs, without CB1R mRNA in VP is indicated by double arrows. Abbreviations; aca, anterior commissure; LHb, lateral habenula; MHb, medial habenula; fr, fasciculus retroflexus.

To identify relevant LHb-projecting afferents, we injected fluorogold (FG) into the medial LHb (Fig. 1A, B). In situ hybridization of CB1R and glutamic acid decarboxylase (GADs, isoforms 65 and 67) transcripts was performed for co-localization in FG immunoreactive (FG-IR) cells projecting to the LHb. Coronal brain sections through the basal forebrain (BF) corresponding to caudal NAcs, and rostral VP were obtained from 3 rats over a span of ~2.0 mm (+2.0 mm to +0.0 mm) rostral to bregma [22]. Moderate FG-IR was found in NAcs beginning from ~+1.4 mm to bregma and extending to ~+0.7 mm (Fig. S1, Supplemental Materials). Lighter FG-IR was also localized to cells in the horizontal and vertical limbs of the diagonal band of Broca (DB, Fig. S1), and some of these DB cells also expressed the cholinergic marker, choline acetyl transferase (ChAT, not shown). FG-IR labeling of cells in NAcs and VP was also noted in more caudal sections, ~0.6–0.9 mm anterior to bregma (Fig. S1). Thus, FG-IR was densest in more caudal regions of the NAcs and the most rostral region of the VP, and some localization was found in the DB. Co-localization of CB1R and GADs mRNAs was observed in 38% of the FG-IR cells in VP (Fig. 1D), and in 18% of the FG-IR NAcs neurons (Fig. 1C–E). These findings are generally consistent with studies describing FG-IR localization after LHb injections in mice and rats [23–26].

#### Synaptic inputs from BF to LHb are primarily GABAergic

**Fig. 2:**
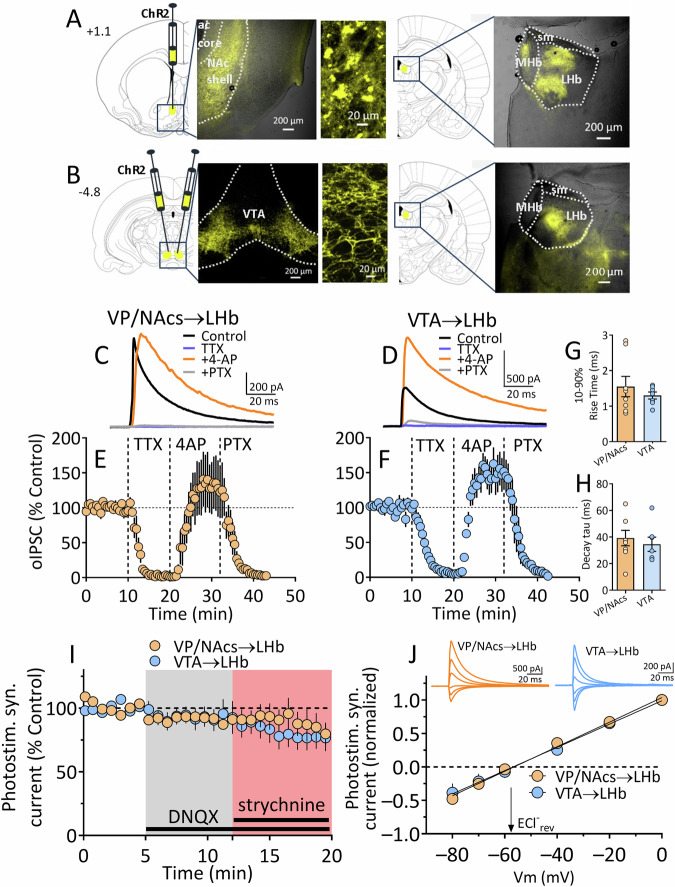
Synaptic currents evoked by ChR2 stimulation in LHb after transfection of VP/NAcs or VTA in are primarily mediated by GABA and not glutamate or glycine. **A** Diagram showing sites of ChR2 construct injection into VP/NAcs and photomicrographs showing eYFP fluorescence at the injection site and in the habenula ~8 weeks after injection. **B** Diagram showing injection sites and eYFP after VTA injections of ChR2 construct. Number at left indicate sections relative to bregma. Mean photostimulation-evoked current sweeps obtained during control periods, and during sequential application of TTX, 4-AP, and PTX in LHb neurons from rats transfected with AAV-ChR2 in the VP/NAcs (**C**) or VTA (**D**). The representative mean time courses for these experiments are shown in (**E**, **F**). In (**G**), the 10–90% rise times are shown for both inputs to LHb, and (**H**) shows the time constant for the decay of the synaptic currents (tau) evoked by each LHb input. These kinetic measures were similar for both pathways (unpaired *t* test; rise time, t_13_ = 0.79, *p* = 0.45, decay time constant, t_13_ = 0.59, *p* = 0.73, respectively). **I** Mean time course for synaptic currents evoked via photostimulation at each pathway during application of the AMPAR antagonist DNQX (10 µM) and glycine receptor antagonist strychnine (5 µM) at a holding potential of 0 mV. **J** Mean current-voltage (I-V) relationships for photoactivated synaptic currents from VP/NAcs and VTA inputs to LHb neurons. The calculated reversal potential for Cl^-^ is indicated by downward arrow. Above are signal averaged synaptic currents collected during activation of each pathway across a range of membrane holding potentials (Vm). The synaptic current I-V curves reversed near that predicted for Cl^-^ ions (ECl^-^, see text). Number of cells/rats: (**E**), 5/3; (**F**), 6/4; (**G–I**), 10/16; (**J**), VTA, 2/3; VP/NAcs, 4/7.

To assess the influence of afferents on LHb neurons in wildtype rats, AAV5-hSyn-ChR2-eYFP was injected into VP/NAcs areas where retrograde FG labeling was observed, or for comparison, into the interfascicular nucleus of the VTA, a mixed GABAergic/glutamatergic LHb input (Fig. 2A, B) [27]. Electrophysiology was conducted in LHb neurons ~8 weeks after transfection. Photostimulation of axon terminals in LHb arising from VTA or VP/NAcs neurons evoked synaptic currents in LHb neurons (V_hold_ = 0 mV) (Fig. 2C, D). The synaptic currents at both inputs were eliminated by the GABA_A_ receptor channel blocker picrotoxin (PTX, Fig. 2C–F). The photoactivated optical IPSCs (oIPSCs) from both projections were also eliminated by the Na^+^ channel blocker, tetrodotoxin (TTX), and this was reversed by the Kv1 K^+^ channel blocker 4-aminopyridine (4-AP) [27], confirming that these currents are monosynaptic (Fig. 2C–F). Additionally, time constants for the rise (Fig. 2G) and decay (Fig. 2H) kinetics of the currents were similar at both pathways (unpaired *t* test; t_13_ = 0.79, *p* = 0.45, and t_13_ = 0.59, *p* = 0.73, respectively). Additionally, the peak synaptic currents evoked at 0 mV membrane potential were not significantly affected by AMPAR (DNQX) or glycine receptor (strychnine) antagonists (Fig. 2I; mixed effects, repeated measures ANOVA, main effect of time, F_2.407, 33.42_ = 3.07, *p* = 0.0512). Similarly, the decay time constants for these currents were unaffected by DNQX or strychnine (Fig. S2), suggesting that glutamate and glycine receptors did not make large contributions to the synaptic currents under these conditions (also see [19]). Membrane voltage versus synaptic current amplitude plots indicated reversal potentials near that predicted for Cl^-^ (E_Cl_) under our recording conditions (Fig. 2J). Thus, considering a calculated liquid junction potential of −9.2 mV, we predicted E_Cl_ = −61.22 mV, and the mean measured E_Cl_ values were: VTA→LHb, E_Cl_ = −56.82 mV, 95% CI = −61.14 to −52.93 mV; NAcs→LHb mean E_Cl_ = −56.47 mV, 95% CI = −59.41 to −53.72 mV; (Fig. 2J). Together, these data show that photoactivation of BF or VTA afferents inhibits LHb neurons via activation of GABA_A_ receptor/Cl^-^ channels.

#### Inhibitory control of LHb neuron excitability by VP/NAcs and VTA inputs to LHb in wildtype rats

**Fig. 3:**
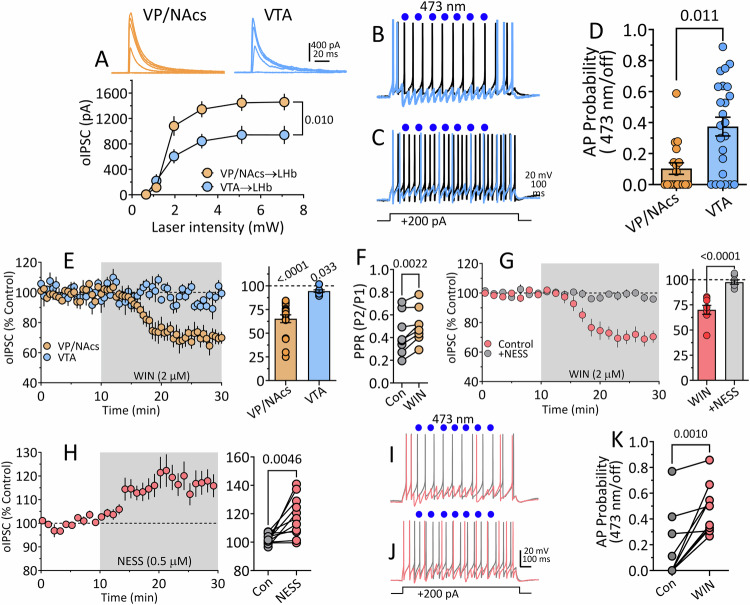
Relative strength of inhibition of LHb neurons by VTA and VP/NAcs afferents and sensitivity to cannabinoids in vitro. **A** Relationship between 473 nm laser power (single -pulses, 2 ms duration) and oIPSC amplitude in LHb neurons from wildtype rats injected with AAV-ChR2 in either VTA or VP/NAcs. VP/NAcs inputs to LHb generate significantly larger oIPSCs than those from VTA. **B**, **C** Action potentials (AP) generated by injection of +200 pA (1 s) currents in LHb neurons. Waveforms in gray were recorded without activation of VP/NAcs or VTA inputs, whereas AP wave forms shown in blue were recorded during stimulation of ChR2 by 473 nm light (blue circles) at VP/NAcs (**B**) or VTA (**C**) inputs (1 s photostimulation train = seven-2 msec duration pulses delivered at 7 Hz at 145 ms intervals during LHb neuron depolarization). **D** Summary of effect of activation of VP/NAcs (*n* = 18) or VTA (*n* = 24) LHb input by ChR2 on the probability of AP discharge. Activation of either input significantly reduced AP probability, but the VP/NAcs input was significantly more effective at silencing LHb neurons t_40_ = 3.526, *p* = 0.011, (unpaired *t* test). **E** Effect of bath application of the cannabinoid agonist WIN55212-2 (WIN, 2 µM) on oIPSCs evoked by ChR2 at VP/NAcs (*n* = 18 cells) or VTA (*n* = 8 cells) inputs to LHb neurons. Whereas WIN had a small effect on IPSCs from VTA input, the effect on VP/NAcs input was significantly larger (*p* = 0.0001, *t* test). The Y-axis title for bar graph is the same as time course. (**F**). WIN significantly increased the paired-oIPSC ratio (PPR) at VP/NAcs inputs to LHb, indicating a presynaptic effect (paired *t* test, t_7_ = 4.7, *p* = 0.022). **G** Preincubation of LHb cells with the neutral CB1R antagonist NESS 0327 (NESS) prevents the inhibition of VP/NAcs oIPSCs in LHb (one-sample *t* test, WIN = t_7_ = 6.76, *p* = 0.0003; WIN + NESS = t_7_ = 1.16, *p* = 0.284). Left and right graph share y-axis. **H** CB1R antagonism by NESS reveals tonic eCB suppression of VP/NAcs oIPSCs in LHb neurons. Left and right panels share y-axis label. **I** AP waveforms generated by current injection with ChR2-activation of VP/NAcs inputs (red) or without ChR2-activation (gray) in control aCSF. **J** AP waveforms recorded with and without ChR2-activation of VP/NAcs inputs to LHb during WIN application. **K** AP probability before (gray circles) and during application of WIN (red circles). The reduction of synaptic inhibition by WIN significantly increased AP probability (paired *t* test, t_9_ = 4.76, *p* = 0.001). Number of cells/rats: **A** VP/NAcs to LHb: 8/5, VTA to LHb, 8/5; (**D**) VP/NAcs to LHb, 18/13, VTA to LHb, 24/16; (**E**) VP/NAcs, 8/6, VTA, 8/5; (**F**). 8/5; (**G**). WIN alone, 8/5: WIN + NESS, 8/5; (**H**) 11/8; (**K**) 10/7.

To determine the influence of these inputs on LHb neuron excitability, we performed whole-cell recordings in brain slices from rats injected with AAV-ChR2 in VP/NAcs or VTA. Plotting the relationship between photoactivated, oIPSC amplitudes and laser intensity, we found that the VP/NAcs inputs to LHb generated significantly larger oIPSCs than those from VTA afferents (2-way RM ANOVA Interaction F_5,90_ = 8.19, *p* < 0.0001; pathway main effect F_1,18_ = 8.231, *p* = 0.01, Fig. 3A).

To examine how these inhibitory inputs control LHb neuron excitability, ChR2 expressed on these axons was photoactivated using brief pulses of light (7 pulses, 145 ms interval), coincidentally with LHb neuron membrane depolarization (1 s, 200 pA) sufficient to generate action potentials (AP) in current clamp (Fig. 3B, C). The probability of AP firing was defined as the number of APs generated by depolarization in the absence of photostimulation, divided by that recorded during photoactivation of the afferents. Although, photostimulation of both LHb inputs reduced AP probability, this was significantly larger for the VP/NAcs BF input compared to that from VTA axons (Fig. 3D, t_40_ = 3.526, *p* = 0.011, unpaired *t* test). Thus, whereas both VP/NAcs and VTA afferents can inhibit LHb neuron excitability, the VP/NAcs input can more effectively silence these glutamate neurons.

### CB1Rs inhibit VP/NAcs input to LHb neurons to control excitability and are tonically activated by eCBs

Our FG-IR experiments showed co-localization of CB1R mRNA in BF cells from VP/NAcs projecting to the LHb (Fig. 1). Therefore, we measured CB1R activation effects on VP/NAcs and VTA synaptic input to LHb. Photostimulated oIPSCs from VP/NAcs inputs were significantly inhibited by the CB1R agonist WIN55,212-2 (WIN, 2 µM; mean inhibition = 65.3% of control, 95% CI = 56.48–74.13% control; one sample *t* test, VP/NAcs = t_17_ = 8.30, *p* < 0.0001). The oIPSCs evoked by photostimulation of VTA inputs were also slightly inhibited by WIN (mean inhibition = 94.56% of control, 95% CI = 91.51 to 97.62% control; one sample *t* test, VTA= t_7_ = 4.30, *p* = 0.004). However, CB1R inhibition of oIPSCs elicited from the VP/NAcs inputs was significantly greater than that at VTA afferents (unpaired *t* test = t_24_ = 4.57, *p* = 0.0001, mean difference = 29.26% control, 95% CI = 16.05–42.47% control, Fig. 3E).

To determine whether the inhibition of oIPSCs by WIN occurred through a presynaptic mechanism on VP/NAcs axons, we measured paired oIPSC responses, evoked by 2 rapid photoactivations of ChR2 (100 ms interval), and calculated the ratio of first to the second oIPSC (paired-pulse ratio, PPR = oIPSC2/oIPSC1). The mean PPR was less than unity before WIN application, suggesting inhibition of the second oIPSC relative to the first, and there was a significant increase in the PPR during WIN application (two-tailed *t* test, t_7_ = 4.7, *p* = 0.022 Fig. 3F). This indicates that WIN decreased GABA release probability from VP/NAcs axons in the LHb. To confirm involvement of CB1Rs, we tested the effect of WIN in brain slices pre-incubated in the neutral CB1R antagonist, NESS0327 (NESS, 500 nM [28]). We found that NESS blocked the inhibition of oIPSCs by WIN (Fig. 3G, WIN alone, mean and 95% CI; 70.05% control, and 60.42–81.47% control; WIN + NESS, 97.57% control 83.37 to 98.52% control; one-sample *t* test, WIN = t_7_ = 6.76, *p* = 0.0003; WIN + NESS = t_7_ = 1.16, *p* = 0.284). This indicates that WIN inhibited VP/NAcs oIPSCs in the LHb by activation of CB1Rs on these axon terminals.

To determine whether endogenous cannabinoids (eCB) might also activate CB1Rs on VP/NAcs axons in LHb, we examined the effect of NESS alone on these responses. We found that NESS significantly increased the size of the oIPSCs, suggesting that they were tonically inhibited by an eCB acting at CB1Rs (Fig. 3H, paired *t* test = t_10_ = 3.64, *p* = 0.0046, mean %control and 95% CI = 115.91% control and 106.156–125.66% control, *n* = 11 neurons).

To determine the consequence of CB1R inhibition of GABA release from VP/NAcs axons on LHb neuron excitability, we next measured the effect of photostimulation of these axons on AP probability before, and during application of WIN. As described above (Fig. 3B, D), ChR2-activation of VP/NAcs inputs strongly inhibited depolarization-induced APs in LHb neurons before WIN was applied (Fig. 3I; mean (±SEM) AP probability = 0.158 ± 0.083, Fig. 3K). However, AP firing probability was significantly increased to 0.479 ± 0.060 during WIN application (Fig. 3J, K, paired *t* test, t_9_ = 4.76, *p* = 0.001, mean difference = 0.3115, 95% CI of difference = 0.1633–0.4597, *n* = 10 neurons). Together, these data show that CB1Rs strongly control GABA release from VP/NAcs afferents to LHb neurons, and that activation of these receptors increases LHb neuron excitatory output.

### LHb Involvement in response inhibition in a food reinforced operant task

**Fig. 4:**
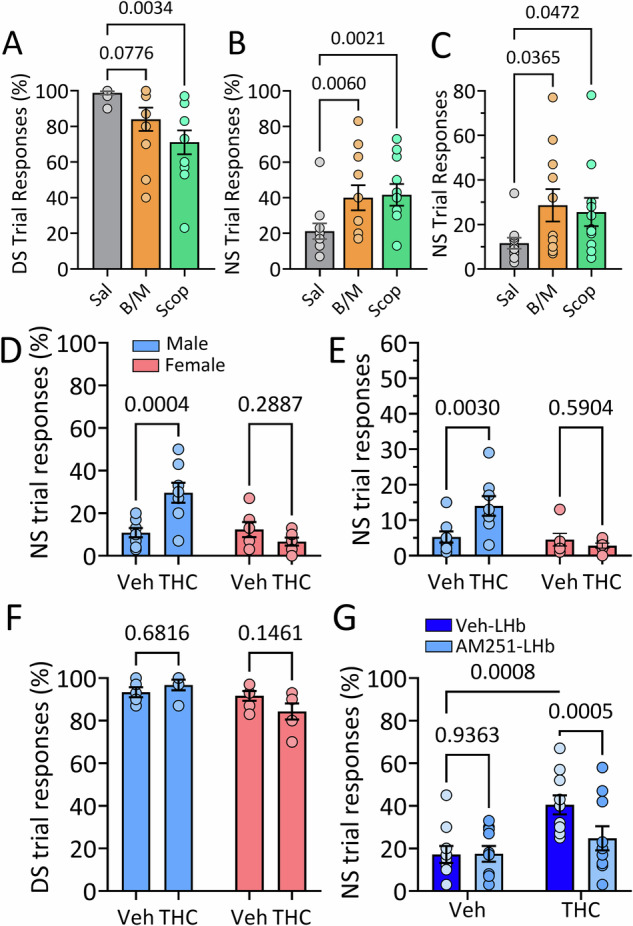
Effects of pharmacological manipulation of the LHb on response inhibition in wildtype rats. **A–C** Effects of intra-LHb infusion of Saline (Sal), baclofen/muscimol (B/M) or scopolamine (Scop) on DS-NS responding (*n* = 8 male and 3 female rats). **A** Scop infusion into LHb significantly decreased responding during trials in which food pellet availability was signaled (DS, repeated measures 1-way ANOVA, F_1.64,16.4_ = 6.95, *p* = 0.009, *p* values from Dunnett’s post hoc test). **B** Scop and B/M significantly increased the percentage of NS trials (when reward was not available) in which responses occurred (repeated measures 1-way ANOVA, F_1.4,14_ = 7.79, *p* = 0.009, *p* values from Dunnett’s post hoc test). **C** Scop and B/M significantly increased the number of NS trial responses (repeated measures 1-way ANOVA, F_1.67,16.73_ = 3.87, *p* = 0.0478, *p* values from Dunnett’s post hoc test). **D–G** Effects of systemic injection of Δ^9^-THC (1 mg/kg) or vehicle on DS-NS responding in male and female rats. **D** Δ^9^-THC injection increased the percentage of NS trials in which responses were observed in male rats only (2-way mixed effects ANOVA, *n* = 8 males and 6 females, drug x sex interaction, F_1,10_ = 12.57, *p* = 0.0053). The numbers above bars in (**D–G**) represent post hoc comparison *p* values using the uncorrected Fisher’s Least Significant Difference test (uFLSD). The legend in (**D**) applies to panels (**D–F**). **E** Systemic Δ^9^-THC effect on the number of responses during NS trials in male and female rats (2-way ANOVA, Drug x Sex Interaction, F_1,24_ = 6.50, *p* = 0.0165). **F** No effect of Δ^9^-THC on percent of trials responding when reward availability was signaled (DS; 2-way mixed effects ANOVA, *n* = 10, F_1,18_ = 3.54, *p* = 0.080). **G** The increase in proportion of NS trial responses caused by systemic injection of Δ^9^-THC in male rats was prevented by infusion of AM251 into the LHb (*n* = 10; 2-Way RM ANOVA, systemic THC x AM251 infusion Interaction = F_1,18_ = 9.35, *p* = 0.0068).

The electrophysiology experiments indicated that BF inputs to LHb strongly inhibit LHb activity, and this inhibition is decreased by CB1Rs. To examine a potential role for this afferent in LHb-involved impulsive behavior, we used a food-reinforced discrimination task that avoided potentially disruptive psychomotor effects of cocaine as a reinforcer [13]. Here, male and female wildtype rats were trained in a DS-NS task using a specific auditory tone (DS) to signal reward availability for responses on an active operant lever. Conversely, white noise, served as a neutral stimulus (NS), indicating no reward for lever presses. Training proceeded until criteria of active lever responding in ≥90% of the DS trials, and ≤30% of NS trials was achieved. To validate LHb involvement in this task, we next examined performance on DS and NS trials during pharmacological and neurobiological manipulations that decreased response inhibition in the 5CSRTT and cocaine-rewarded Go/NoGo paradigm [13–15]. Infusion of scopolamine into LHb significantly decreased the percentage of DS trials in which responding was observed, whereas infusion of B/M did not significantly alter this measure (Fig. 4A). However, these pharmacological manipulations did not alter the number of responses on the inactive operant lever (Fig. S3A) or the number of food port entries (Fig. S3B). Importantly, like our previous studies, B/M, or scopolamine infusion into LHb significantly increased NS trials in which responding was observed, whether analyzed as percent of NS trials, or total responses during NS presentation (Fig. 4B, C).

In a previous study, we also found that response inhibition in the 5CSRTT and Go/NoGo tasks was impaired by systemic injection of Δ^9^-THC (1 mg/kg) [15], and our present study and others show that some LHb afferents express CB1Rs [18, 19, 29, 30] (Fig. 1). Therefore, we examined the effect of Δ^9^-THC on DS-NS behavior and found that response inhibition was significantly impaired by systemic injection of the phytocannabinoid, although this was observed in only male rats (mixed effects ANOVA, drug x sex interaction F_1,10_ = 12.57, *p* = 0.0053, Fig. 4D, E). In addition, Δ^9^-THC did not alter the number DS trials in which responding was observed in males or females (F_1,18_ = 3.454, *p* = 0.0795, mixed effects ANOVA, Fig. 4F). However, we did observe a significantly higher number of inactive lever responses in males compared to females (Fig. S3C), but this measure was not significantly altered by systemic Δ^9^-THC (Fig. S3C). Our prior studies also showed that infusion of the CB1R antagonist AM251 into LHb prevented disruption of response inhibition by systemic Δ^9^-THC in the 5CSRTT and Go/NoGo paradigms [15]. Similarly, intra-LHb AM251 also blocked the pro-impulsive effects of systemic Δ^9^-THC in the present DS-NS task in male rats (Fig. 4G, *n* = 10; 2-Way RM ANOVA, Interaction = F_1,18_ = 9.35, *p* = 0.0068, p = 0.0005, Uncorrected Fisher’s Least Significant Difference (uFLSD) post hoc). Therefore, similar to our previous measures of impulsivity, response inhibition in the DS-NS paradigm requires intact LHb function and is impaired by disruption of LHb cholinergic signaling or by systemic injection of Δ^9^-THC.

### VP/NAcs projections to LHb influence response inhibition in the DS-NS task in GADCre rats

**Fig. 5:**
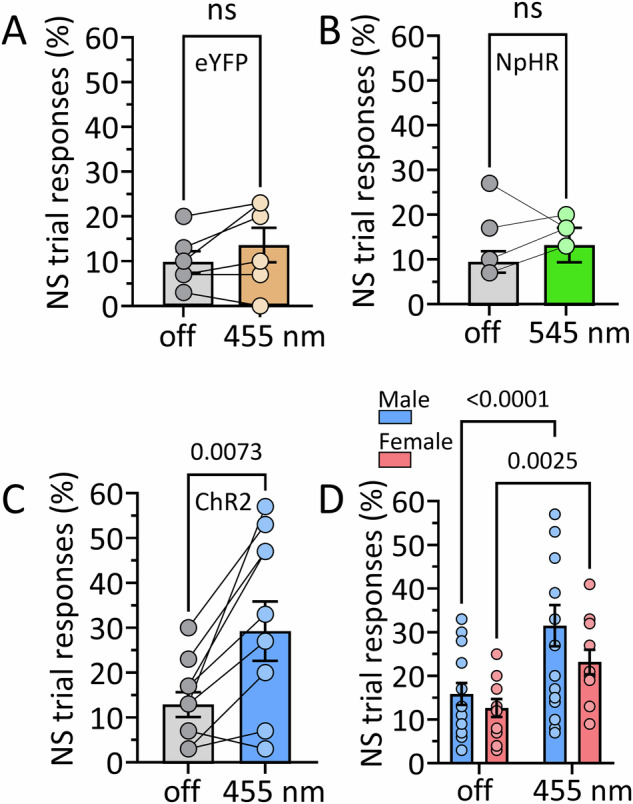
Impaired response inhibition during activation of GABAergic BF input to the LHb by ChR2 in GADCre rats. **A** Comparison of the percent of neutral stimulus (NS) trials in which responses on the active lever were observed in the absence (off) and presence of 455 nm light delivered via optical fibers terminating above the LHb, ~8 weeks after expression of Cre-dependent eYFP in the VP/NAcs (*n* = 6 rats; paired *t* test, t_5_ = 1.67, *p* = 0.155). **B** Optogenetic inhibition of VP/NAcs GABAergic inputs to LHb by stimulation of NpHR with 545 nm light does not affect NS responding (*n* = 4 rats; paired *t* test, t_3_ = 0.38, *p* = 0.728). **C** Activation of VP/NAcs GABAergic inputs by light stimulation of ChR2 in LHb significantly increased the percent of NS trials in which responses were observed (paired *t* test; *n* = 10 male rats, t_9_ = 3.44, *p* = 0.007). The total number of NS responses shown in Fig. S4A. **D** ChR2 photostimulation with 455 nm light significantly increases responding during presentation of the NS, and this is not significantly different between male and female GADCre rats (*n* = 16 male and 11 female rats; 2-way RM ANOVA, light x sex interaction, F_1,25_ = 1.546, *p* = 0.225; main effect of light, F_1,25_ = 41.03, *p* < 0.0001; Post hoc comparisons by uFLSD, effect of light in males, *p* < 0.0001; effect of light in females, *p* = 0.0025). ns, non-significant.

To determine whether the GABAergic inputs to LHb arising from the VP/NAcs influence response inhibition, we trained GADCre rats [20] in the DS-NS paradigm, 7–8 weeks after infusion of viruses expressing the inhibitory halorhodopsin (AAV- EF1α-DIO-eNpHR3.0-eYFP (NpHR)), excitatory Channelrhodopsin-2 (AAV5-hSyn-ChR2-eYFP, ChR2), or enhanced yellow fluorescent protein alone (AAV-Control; AAV5- hSyn1-eYFP) into the VP/NAcs. We then assessed the effects of photostimulation of VP/NAcs axon terminals in the LHb (NpHR, 545 nm, constant pulse, alternating 5 s on/off; ChR2 and eYFP, 455 nm, 60 Hz pulses, alternating 5 s on/off) on operant responding during DS and NS trials. Photostimulation of the LHb failed to alter NS responding in rats receiving eYFP (Fig. 5A) or NpHR constructs into the BF (Fig. 5B). However, photoactivation of ChR2 in the LHb significantly increased the percentage of NS trials in which responses were observed (Fig. 5C, D), as well as the total number of these responses (Fig. S4A) in GADCre rats. This impairment in response inhibition caused by photostimulation of ChR2 on this pathway did not significantly differ between male and female rats (Fig. 5D; *n* = 16 male and 11 female rats; 2-way RM ANOVA, light x sex interaction, F_1,25_ = 1.546, *p* = 0.225; main effect of light, F_1,25_ = 41.03, *p* < 0.0001; Post hoc comparisons by uFLSD, effect of light in males, *p* < 0.0001; effect of light in females, p = 0.0025). In contrast, photostimulation of ChR2 in LHb did not significantly affect responding during DS trials (Fig. S4B), the number of food port entries (Fig. S4C), nor the number of responses on inactive levers (Fig. S4D). Similar results were observed in optogenetic experiments in wildtype rats (Fig. S5). The data suggest that increased synaptic inhibition of LHb neurons via stimulation of VP/NAcs GABAergic axons increases impulsivity, as measured by decreased response inhibition in the DS-NS task.

## Discussion

Impulsive action, defined by the inability to suppress a previously learned response, is a neurocognitive measure sensitive to alterations in brain states associated with stroke, psychiatric illness, and drug use [1, 2, 11, 31, 32]. Moreover, trait impulsivity is a significant risk factor for the development of SUDs [8]. Using impaired response inhibition as a surrogate of impulsivity during cocaine seeking, we reported previously that inactivation of LHb neurons impaired inhibition of responding for the psychostimulant [13, 14]. Similarly, blockade of LHb M_2_ mAChRs [13, 14], or systemic injection of Δ^9^-THC also impaired response inhibition in Go/NoGo cocaine seeking [15]. Additionally, systemic cocaine or Δ^9^-THC dose-dependently increased impulsivity in the 5CSRTT, without altering measures of attention [15]. Importantly, in this latter study the effects of cocaine and Δ^9^-THC were prevented by LHb CB1R antagonism [15], suggesting that the LHb eCB system is engaged by both drugs and is involved in the loss of response inhibition.

Here, using retrograde FG labeling, we characterize a GABAergic input to the LHb that originates in the BF, with neurons located in rostral VP, caudal NAcs, and DB, that co-localize GAD and CB1R mRNA. Previous studies identified projections from rostral and ventromedial areas of the VP to the medial LHb [26, 33], and GABAergic and glutamatergic VP afferents to LHb in mice [23, 24]. However, the proportion of VP cells localizing the type 2 vesicular glutamate transporter (VGluT2) was much lower, and the proportion of GAD positive GABAergic cells much higher in the rostral versus the caudal VP [24]. Additionally, there are several neuroanatomical reports of LHb-projecting neurons that originate in the NAcs [26, 34–36].

Once identified, we targeted this area for viral expression of ChR2 to measure its synaptic properties in LHb during photoactivation of this pathway. These experiments confirmed that the majority of the LHb projecting VP/NAcs neurons transfected by AAV-ChR2 were GABAergic. Moreover, a comparison of the influence of VP/NAcs afferents and GABAergic inputs from VTA neurons on LHb neurons showed that the former pathway generated larger oIPSCs that more effectively silenced LHb neuron action potential discharge. Assuming similar efficiencies of transfection with AAV-CHR2, this implies that whereas both VTA and VP/NAcs afferents can inhibit LHb neurons, the VP/NAcs input likely exerts particularly strong inhibition of this brain area.

The co-localization of FG-IR and CB1R mRNA to VP and NAcs neurons suggests that this is a likely substrate for eCB modulation of LHb function. Consistent with this, comparison of photostimulated oIPSCs from VP/NAcs with VTA inputs to LHb showed that those from VP/NAcs were more strongly inhibited by a CB1R agonist. Additionally, the neutral CB1R antagonist, NESS0327, increased the amplitudes of oIPSCs evoked from VP/NAcs inputs to LHb, indicating that GABA release from this afferent is under tonic inhibitory control by an eCB. Previous studies identify both 2-arachidonoylglycerol (2-AG) and anandamide in the LHb [18, 19], but only manipulation of 2-AG levels altered synaptic GABA release in LHb neurons [19]. Moreover, Berger et al (2018) propose that stress-induced 2-AG release activates CB1Rs in LHb to reduce GABAergic input, leading to increased excitation of LHb neurons. Our present data are generally consistent with this hypothesis, and we identify a GABAergic BF afferent that could underlie this stress response. Whereas the inhibition of VP/NAcs inputs to LHb by CB1Rs is a property shared with lateral preoptic area GABAergic and glutamatergic inputs to LHb [19], our demonstrated insensitivity of VTA afferents to CB1R activation is shared with inhibitory entopeduncular nucleus inputs to the LHb [19]. Taken together, these studies indicate that LHb afferents are not uniformly modulated by cannabinoids, with pathway-selectivity determined by presynaptic CB1Rs, and this likely provides a substrate for eCBs to influence LHb-dependent behavior.

To evaluate a potential role for VP/NAcs inputs to the LHb in impulsive behavior, the present study used food reward in a DS-NS discrimination paradigm to measure response inhibition. Like our previous studies with Go-NoGo cocaine reward [13, 14], we found that inhibition of LHb neurons, blockade of LHb mAChRs, or systemic Δ^9^-THC exposure [15], all increased responding during signaled non-reward trials. Thus, our results indicate that response inhibition for cocaine or food reward is impaired by altered LHb function, and the present results further suggest that psychostimulant interaction with LHb relevant circuitry is not prerequisite to its involvement in impulsive behavior. Moreover, unlike premature responding in the 5CSRTT, where LHb might be recruited via punishment relating to delayed reward, there is no apparent punishment for responding during unrewarded NS trials in the DS-NS paradigm. Our present results also show that although photoactivation of BF input to LHb impaired inhibition of responding for food reward, this did not affect food-port nose pokes, responses during DS trials, or inactive lever responses, suggesting that BF pathway activation did not alter reward salience, motivation to seek reward, or cause a general increase in motor activity. Together, the results imply that intact LHb function is necessary to maximize reward efficiency by participating in the suppression of unproductive responding.

Like its effects on impulsivity in the 5CSRTT [15], here we found that Δ^9^-THC decreased response inhibition in the DS-NS task. However, this was observed in male and not female rats (Fig. 4D, E). As only male rats were included in this previous study [15], we cannot predict whether the insensitivity to Δ^9^-THC in females in the DS-NS task generalizes to other measures of impulsivity. However, impaired response inhibition upon photostimulation of ChR2 on the VP/NAcs projection to LHb was seen in both sexes (Fig. 5D), suggesting that the insensitivity to Δ^9^-THC in females likely does not result from differences in the strength of inhibitory input to LHb, as previously reported in male and female mice [37]. Instead, as another study shows sex-dependent differences in levels of 2-AG in LHb, as well as differential sensitivity to CB1R control of stress coping strategy selection [18], it is more likely that our Δ^9^-THC results may be explained by eCB system differences rather than by sexual dimorphism in the density of inhibitory afferents targeting LHb.

Our previous data showing that direct LHb inhibition increases impulsivity, and our present results showing that photoactivation of inhibitory input to the LHb also impairs response inhibition, indicate that suppression of LHb activity impairs impulse control. Consistent with this, we found that NpHR inhibition of VP/NACs LHb afferents did not alter response inhibition, suggesting that this pathway may not be engaged in DS-NS task performance under the present conditions. Alternatively, the indiscriminate inhibition of CB1R and non-CB1R expressing GABAergic neurons in GADCre rats may obscure a behavioral effect of inhibiting the CB1R-expressing axons alone. However, the lack of NpHR effect, together with the impairment of response inhibition by ChR2, suggests that activation of this LHb input is sufficient to increase impulsivity, but is not necessary for the execution of DS-NS behavior. Although the circumstances under which this pathway is recruited during behavior are largely unknown, it is possible that the VP/NAcs input to LHb may be involved in other LHb-relevant behaviors.

The lack of NpHR effect on DS-NS responding also makes it unlikely that the increase in impulsivity observed with systemic Δ^9^-THC occurs through suppression of GABA release by CB1Rs on this BF pathway. Therefore, although we find that LHb CB1R antagonism consistently blocks the pro-impulsive effects of systemic Δ^9^-THC in distinct behavioral tasks [13, 14], it remains unlikely that the pro-impulsive effects of the phytocannabinoid occur through suppression of a GABAergic input to LHb. Alternatively, CB1R inhibition of glutamate release from unidentified LHb afferents, and from lateral preoptic area inputs have been reported [19, 30]. Therefore, Δ^9^-THC may act upon this alternative mechanism to contribute to heightened impulsivity.

Although, at present the circumstances leading to recruitment of this cannabinoid modulated BF input to LHb are unknown, we show that this pathway is highly sensitive to CB1R activation and is tonically inhibited by an eCB in vitro. This suggests that that it may be involved in other behaviors regulated by the eCB system. In this regard, LHb levels of 2-AG are increased by acute and chronic stress, and LHb CB1Rs contribute to selection of strategies to cope with stressful situations [18]. Moreover, anxiogenic response to high-doses of synthetic cannabinoid agonists depend upon expression of CB1Rs on GABA neuron axon terminals in mice, as this is abolished by deletion of these receptors on GABA cells [38]. Another behavior that may be regulated by the LHb eCB system acting on VP/NAcs inputs is that of rodent inter-male aggression reward [26]. In this model, photoactivation of BF GABAergic inputs to LHb was sufficient to increase reward associated with defeat of subordinates in socially dominant mice [26]. As there appears to be overlap between the LHb projecting BF regions targeted in this previous study and our present one, it is possible that the effect of cannabinoids on this pathway that we report may modulate social defeat reward. In general, more studies with additional behavioral models are necessary to identify roles for specific inputs to the LHb, and to define how modulation of these inputs by the LHb eCB system may be involved.

## Supplementary information

Supplemental Methods and Figures

## Data Availability

All data analyzed in this study are included in this published article
