# Supplementary material for: Basal forebrain-lateral habenula inputs and control of impulsive behavior
Source: Neuropsychopharmacology. 2024 Aug 18;49(13):2060–8. doi: 10.1038/s41386-024-01963-7 (PMC11480124; doi:10.1038/s41386-024-01963-7)
Supplement: Supplementary file 1 — Supplemental Methods and Figures [file 41386_2024_1963_MOESM1_ESM.docx]

**Supplemental Materials**

**Methods**

*Viral construct injections.* For electrophysiological experiments, a 10-µl Hamilton syringe (35 gauge needle) was connected to a UltraMicroPump/SYS-Micro4 controller (WPI) to deliver 0.7 µl of AAV5-hSynhChR2(H134R)-eYFP (University of North Carolina Vector Core), at 0.1 µl/min, into the VP/NAcs (all coordinates from bregma, midline, and skull surface (in mm): AP: +1.6; ML: ± 0.8; DV: -6.8), or the medial VTA (AP: -5.4; ML: ± 0.2; DV -8.2, 8° angle) in Wildtype Long Evans rats. For behavioral experiments, GADCre rats received one of three viral constructs (Addgene) infused bilaterally (0.3 µl at 0.150 µl/min) in the VP/NAcs area (coordinates; AP + 1.5; ML ± 0.9; DV -7.0); AAV5-EF1a-dio-eYFP (eYFP, Catalog # 26966-AAV5), AAV5-EF1a-dio-NpHR 3.0h-eYFP (NpHR, Catalog # 26966-AAV5) or AAV5-EF1a-dio-hChR2-eYFP (ChR2; Catalog #20298-AAV5). Injection needles were left in place for 2 min after injection, and then slowly withdrawn.

*Infusion cannulae and optical fiber implantation.* Rats were implanted under isoflurane anesthesia with bilateral guide cannulae (C315, Plastics One, Roanoke VA), positioned 1 mm above the LHb (AP: −3.8, ML: ±0.6, DV: −3.6 mm). Rats in optogenetic experiments received optical fibers (200 µm, 0.66 NA) positioned above the LHb (AP: −3.6, ML: ±1.2, DV: −4.8 mm, 6° angle).

*Pathway tracing.* Fluorogold (FG; 1% in cacodylate buffer, pH 7.5) was delivered iontophoretically through a stereotaxically positioned glass micropipette (inner tip diameter 30-40 µm) by applying 1µA current in 7 s pulses at 14 s intervals for 10 min, into the medial portion of the LHb (AP -3.4 mm, ML ± 0.8 mm, DV -5.0 mm) of 3 male Long-Evans rats (330–360 g). The injections were unilateral, and the micropipette remained in place for 10 min to prevent backflow. One week following FG injection, rats were anesthetized and perfused transcardially with 4% paraformaldehyde (PF) in 0.1 M phosphate buffer (PB), pH 7.3. The brains were removed and placed in 4% PF for 2 h, then transferred to 18% sucrose in PB overnight at 4 °C. Coronal serial cryosections containing the BF (~+2.0mm to 0.0 mm) were prepared for immunohistochemistry and RNAscope in situ hybridization-immunohistochemistry.

*FG detection by immunolabeling.* Free-floating coronal sections were placed in PB (1h) supplemented with 4% BSA and 0.3% Triton X-100, followed by incubation overnight (at 4℃) with primary rabbit anti-FG antibody (1:1000, AB1531, MillporeSigma, Burlington, MA). After PB rinsing 3 x 10 min, slices were incubated in biotinylated goat anti-rabbit antibody (1:200, Vector Laboratories) for 2h at room temperature. The sections were then rinsed with PB and incubated for 1h at room temperature in avidin-biotinylated horseradish peroxidase (1:100, ABC kit, Vector Laboratories). Sections were then rinsed in PB, and the peroxidase reaction developed with 0.05% 3,3’-diaminobenzidine (DAB) and 0.03% H_2_O_2_. Sections were mounted on coated slides, and bright-field images were collected with an Olympus MVX10 and a 63x objective (Olympus, Waltham, MA).

*FG colocalization with RNAscope and immunolabeling.* Serial coronal cryosections (16 μm) were used to colocalize FG immunoreactive (FG-IR) neurons with mRNA using RNAscope in situ hybridization-immunolabeling. Brain sections were incubated (30°C, 2h) with rabbit anti-FG antibody (1:200, AB1531, MillporeSigma, Burlington, MA), in DEPC-treated PB with 0.5% Triton X-100, containing RNasin (Promega, Madison, WI). Sections were rinsed 3 x 10 min DEPC-PB, and incubated (1h, 30°C) with secondary Alexa Fluor 750 anti-Rabbit antibody (1:100, ab175728, Abcam, Boston, MA). Sections were rinsed with DEPC-PB, mounted onto Fisher SuperFrost slides and dried overnight at 60°C, followed by RNAscope in situ hybridization using the manufacturer’s instructions. Briefly, sections were treated with heat and protease digestion followed by hybridization with a cocktail of probes for detection of transcripts encoding glutamic acid decarboxylases (GADs; GAD1 316401-C2; GAD2 435801-C2, Alexa 488, Advanced Cell Diagnostics, Newark, CA) and the CB1R (412501, Atto 550, Advanced Cell Diagnostics, Newark, CA). Additional sections were hybridized with the bacterial gene DapB as a negative control. RNAscope in situ hybridization and immunolabeled sections were viewed and photographed with a Zeiss confocal microscope with Airyscan/CY7.5 (LSM880, Zeiss, White Plains, NY). Negative control hybridizations showed negligible fluorophore expression. Neurons were counted when a FG-IR cell was at least 5 μm in diameter, and photomicrographs were adjusted to match contrast and brightness using Adobe Photoshop (Adobe Systems, San Jose, CA) and GIMP (v2.10.36).

*Behavioral training.* Rats were trained on a discriminative stimulus-neutral stimulus (DS-NS) paradigm adapted from Ambroggi et al. [1]. At least one week after surgery, rats were food restricted to 90-95% of their free feeding weight (12-15 grams of lab chow per day). Training and testing occurred in standard Med-Associates (Fairfax, VT) rat operant boxes equipped with two cue lights located above two retractable levers, with a food magazine located between the levers. A house light, a white-noise generator, and a tone generator were located on the opposite wall. Initially, rats were trained to press a randomly designated active lever. Each press (FR1) on the active lever delivered one 45 mg food pellet (Bio-Serv, Flemington, NJ), followed by retraction of both levers, switching off the house light, and switching on of the cue-light (for 5 seconds) located above the active lever. Responses on inactive levers did not have consequences. After 40 pellets were collected during a 30 min trial for at least 3 consecutive days, training was begun on the DS-NS paradigm. During this training, a 10 second tone (4.5 KHz, DS) or white noise (NS) was presented randomly on a variable interval (VI) schedule at 10 dB above baseline noise. Training progressed using intervals of 10, 30, and 60 seconds, with the number of stimulus presentations equal to 200, 100, or 60, respectively. The final schedule consisted of 30 DS and 30 NS stimulus presentations at a VI60. Responses during the DS resulted in the reinforcement sequence experienced during FR1 training, and responses during the NS or in the absence of stimuli were never reinforced. Training proceeded until the proportion of stimulus presentations to which the animal responded was at least 90% for the DS and lower than 30% for the NS. Unlike DS trials, in which a single response on the active lever ends the trial, in NS trials, multiple responses can be recorded. Therefore, both the percent of NS trials with responses, as well as the actual number of responses are shown for NS trials.

*Intracranial drug infusion.* Baclofen and muscimol, injected together (B/M, 1 and 0.1 mM, respectively), or scopolamine (50 mM), were dissolved in sterile saline prior to bilateral injection into the LHb. The infusions were performed using a microinjection cannula (C315I, Plastics One) inserted into the surgically implanted guide cannula and extended 1.0 mm below its tip. Then, one of the solutions (0.5 μl) was infused at a rate of 1 μl/min. The microinjection cannula was removed 1 min after the infusion, and the animals immediately placed in the operant boxes. For AM251 (Tocris), the drug was dissolved in DMSO (50 mM stock) and diluted to 2.5 mM (final vehicle 5% DMSO, 2.5% Tween80, 92.5% Saline). Immediately after the AM251 or vehicle infusion, rats received a single intraperitoneal injection of either Δ^9^-THC (1mg/kg) or vehicle (20% DMSO, 10% Tween80, 70% Saline), then returned to their home cage for 20 minutes before being placed in the operant boxes. Rats were tested using a within subject design. Each animal received the different treatments sequentially in a pseudo-random manner, allowing at least 48 hours between consecutive drug test days.

*In vivo optogenetics.* At least 8 weeks after AAV infusions, an LED source (Prizmatix, Southfield, MI) was connected to the implanted fiber optic cannula via an optic fiber tether connected to a rotary joint located on the ceiling of the operant chamber. Rats were habituated to perform the task while connected to the tether for at least five sessions prior to optogenetic manipulations. On test days, the LED delivered light (5-10 mW at the cannula tip) continuously (545 nm, NpHR), or via 60 Hz pulses (455 nm, ChR2, eYFP) throughout the testing session (alternating 5 seconds ON/OFF).

*Brain slice preparation.* Wildtype rats were anesthetized with isoflurane, followed by transcardial perfusion with oxygenated (95% O_2_/ 5% CO_2_), ice-cold (2-4 ℃), artificial cerebrospinal fluid (aCSF), (in mM): N-Methyl-D-Glucamine (NMDG) 93, KCl 2.5, NaH_2_PO_4_ 1.2, NaHCO_3_ 30, HEPES 20, glucose 25, sodium ascorbate 5, sodium pyruvate 3, MgCl_2_ 10, CaCl_2_ 0.5 (pH 7.4, 300-310 mOsm). After perfusion, rats were decapitated using a guillotine and the brain removed into ice-cold aCSF. Coronal brain slices (250 μm) were then prepared using a vibrating tissue slicer (Leica VT1200S, Heidelberg, Germany) according to previously published protocols [2]. Slices were incubated in the above NMDG-aCSF solution at 34 °C for 10-15 min, and then transferred to a holding chamber containing modified aCSF consisting of (in mM): NaCl 109, KCl 4.5, MgCl_2_ 1, CaCl_2_ 2.5, NaH_2_PO_4_ 1.2, NaHCO_3_ 35, glucose 11, HEPES 20, sodium ascorbate 0.4 (pH 7.4, 300-310 mOsm), at room temperature. For recording, a single brain slice was submerged in a recording chamber (~300 µL) integrated into a fixed stage of an upright microscope, and continuously perfused with oxygenated aCSF (2-3 ml/min), containing (in mM); NaCl 126, KCl 3.0, MgCl_2_ 1.5, CaCl_2_ 2.4, NaH_2_PO_4_ 1.2, glucose 11.0, NaHCO_3_ 26. The aCSF was maintained at 30-32 °C using an in-line heater (TC-324B, Warner Instruments, Hamden, CT).

*LHb neuron recordings in vitro.* LHb neurons were visualized using a 40X water immersion objective, and a gradient contrast microscope with infrared illumination, connected to a digital video monitor (Olympus BX51WI, Tokyo, Japan). Whole-cell voltage clamp recordings were performed with borosilicate glass electrodes (3 to 4 MΩ), filled with an internal solution containing (in mM); 117.5 CsMeSO_3_, 10 HEPES, 10 TEA-Cl, 8 CsCl, 0.25 EGTA, 5 QX-314 (Br), 10 Na-Phosphocreatine, 4 Mg-ATP, 0.3 Na-GTP; adjusted (pH 7.3, 290-300 mOsm). For current clamp recordings, recording pipettes were filled with an internal solution containing (in mM); 140 K-gluconate, 10 HEPES, 0.2 EGTA, 2 MgCl_2_, 0.1 CaCl_2_, 4 Na_2_-ATP, 0.3 Na-GTP (pH7.3, 280-290 mOsm). All recordings were performed using a Multiclamp 700B amplifier (Molecular Devices, San Jose, CA), WinLTP software (WinLTP Ltd; www.winltp.com), and an A/D board (National Instruments PCI-6251, Austin, TX) residing in a personal computer. Series resistance was monitored using brief hyperpolarizing steps (-5 mV, 200 ms), was generally 10-20 MΩ, and was not compensated. Cells demonstrating >10% change in series resistance during the recording were excluded from the study. Unless otherwise indicated, cells were voltage clamped at 0 mV to measure GABAergic currents. Unless otherwise stated, photostimulated optical IPSCs (oIPSCs) were obtained every 30 s (0.033 Hz) using 473 nm laser light (2 ms) that was fed into an optical fiber and collimated through the 40x microscope objective using an IS-OGP/OLY adapter (Siskiyou Corp., Grants Pass, OR). To isolate oIPSCs, LHb neurons were held at 0 mV and DNQX (10 μM) and DL-AP5 (100 μM) were added to the aCSF to block glutamatergic synaptic responses. Input-output (I-O) relationships between evoked synaptic current amplitude and laser stimulus intensity (0.7, 1.1, 2.0, 3.3, 5.1, 7.1 mW/mm^2^) were constructed for LHb afferents. To monitor presynaptic changes, the ratios of pairs of oIPSCs (paired-pulse ratio; PPR) were collected using two consecutive laser stimuli with a 75 ms inter-stimulus interval (ISI). The ratio was calculated by dividing the peak amplitude of the second oIPSC (P2) by the first oIPSC (P1). The ratios of 4 consecutive responses were averaged for each cell in each condition. In current clamp recordings, LHb neuron excitability was measured by evoking action potentials using 1s, 200 pA current injected through the patch electrode. Action potential (AP) probability was defined as the number of action potentials elicited in the absence of photostimulation, divided by the number of action potential measured during photoactivation of ChR2-AAV transfected axon terminals (seven-2 msec duration pulses delivered at 7 Hz with 145 ms intervals). Calculated Cl^-^ reversal potentials were corrected for liquid junction potentials defined by the stationary Nernst–Planck equation [3], using LJPcalc software (<https://swharden.com/LJPcalc>).

*Data Analysis.* In voltage clamp experiments, representative traces are the averages of 10 consecutive responses. Data tables, graphing and statistical analyses were compiled with GraphPad Prism 10.0 (GraphPad Software Inc., USA) and Clampfit (version 9, Molecular Devices). Data are presented as mean ± standard error of the mean (SEM). Statistical significance was determined using one-way, two-way, or mixed-effects repeated measures ANOVA, and appropriate multiple-comparison post hoc tests were conducted only after significant main effect was observed, and the significance level was P<0.05. Unless stated, “n” refers to the number of neurons from which recordings were made. However, the number of neurons recorded from the number of rats (n/R) is reported in the figure captions summarizing each experiment.

*Reagents.* DL-AP5, 6,7-Dinitroquinoxaline-2,3-dione (DXQX), DL-2-Amino-5-phosphonopentanoic acid (DL-AP5), Picrotoxin, (R)-(+)-WIN 55212, scopolamine and AM251 were purchased from Tocris Bioscience (Minneapolis, MN). NESS 0327 was purchased from Cayman Chemical (Ann Arbor, MI, USA), and Δ^9^-THC was obtained from the National Institute on Drug Abuse drug supply (Rockville, MD). The drugs were delivered to brain slices via the perfusion system in aCSF via gravity and were prepared as stock solutions in H_2_O or DMSO and Tween 80 and diluted to indicated concentrations.

**
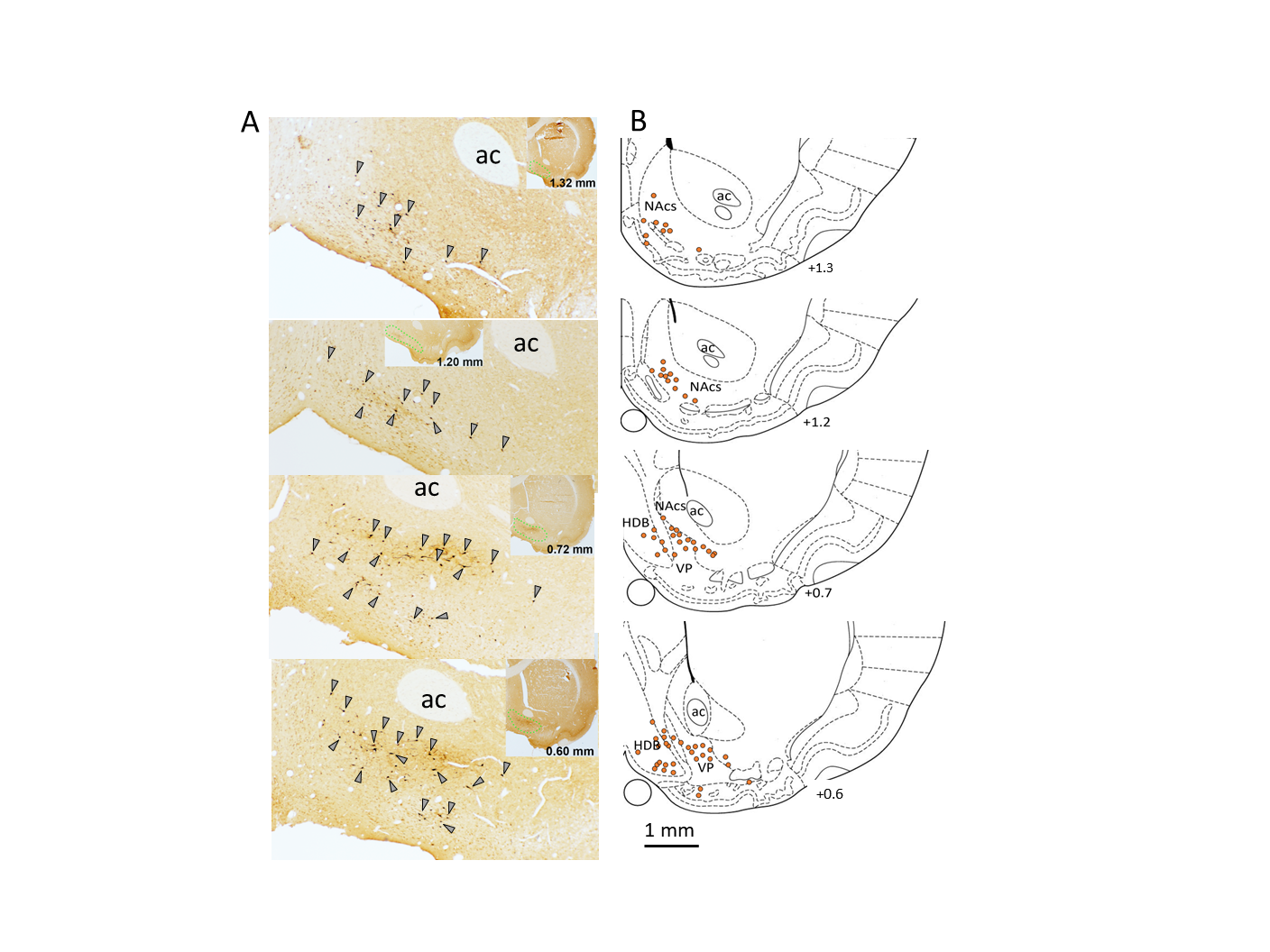
Supplemental Figures**

**Supplemental Figure 1.**  Light microscope localization of retrograde fluorogold immunoreactivity (FG-IR) in the forebrain after injections into the medial lateral habenula in a single rat. **A.** Histology showing FG-IR in individual neurons in brain sections corresponding to +1.32 to +0.6 mm anterior to bregma. In each section the anterior commissure (ac) is indicated. Insets show the same section at lower microscope power. Gray arrow heads indicate positions of some FG-IR neurons and indicating the lateral-medial extent of labeling. **B**. Approximate positions of FG-IR labeled neurons (orange dots ) in brain atlas diagrams corresponding to those shown in **A** [4]. Abbreviations: NAcs, nucleus accumbens shell; VP, ventral pallidum; HDB, horizontal diagonal band of Broca; ac, anterior commissure. Scale bar in **B**, corresponds approximately to that shown in panel **A**.


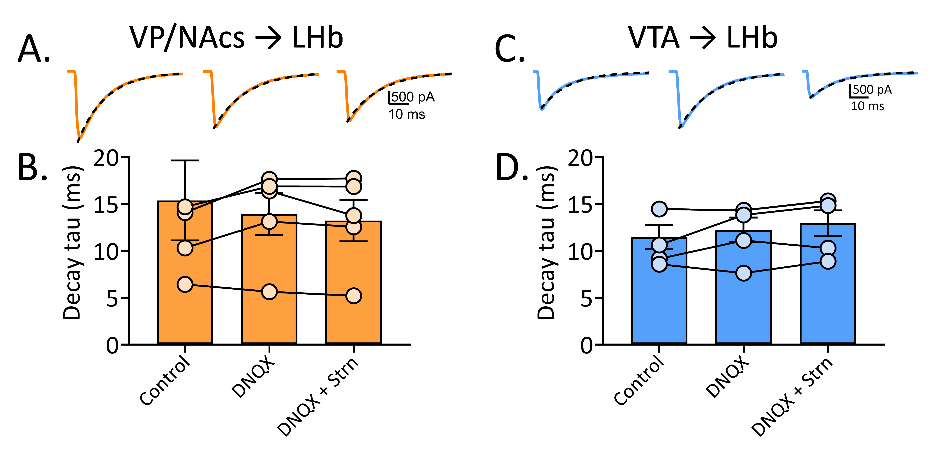


**Supplemental Figure 2.** Data from same experiment shown in Fig. 2I. Effects of DNQX and strychnine (Strn) on decay kinetics of photoactivated synaptic currents recorded in voltage clamped LHb neurons (0mV holding potential) receiving input from either of the indicated projections. **A.** Signal-averaged synaptic currents evoked from VP/NAcs axons, collected during the indicated sequential application of antagonists. The decay of each waveform was fit with a one-phase exponential function (black dashed line) to estimate the decay time constant, tau (τ), or the value at which the synaptic current has decayed to 63.2% of its peak value. **B.** Decay values measured in individual cells (n = 5 cells), together with mean and SEM for the same data. **C.** Waveforms and curve fits of synaptic currents evoked by photostimulation of VTA axons in the LHb. **D.** Decay vales from each cell and group means for VTA to LHb inputs (n = 5 cells). One-way repeated measures ANOVAs were conducted for data shown in **B**. and **D**, and neither was significant (VP/NAcs, F_1,4_ = 0.2594, p = 0.6376; VTA, F_1,4_ = 2.682, p = 0.1567). Additionally, there was no significant difference between decay time constants at these pathways (t­_8_ = 0.8808, p = 0.4041, unpaired t-test comparison of control data from **B** and **D**).


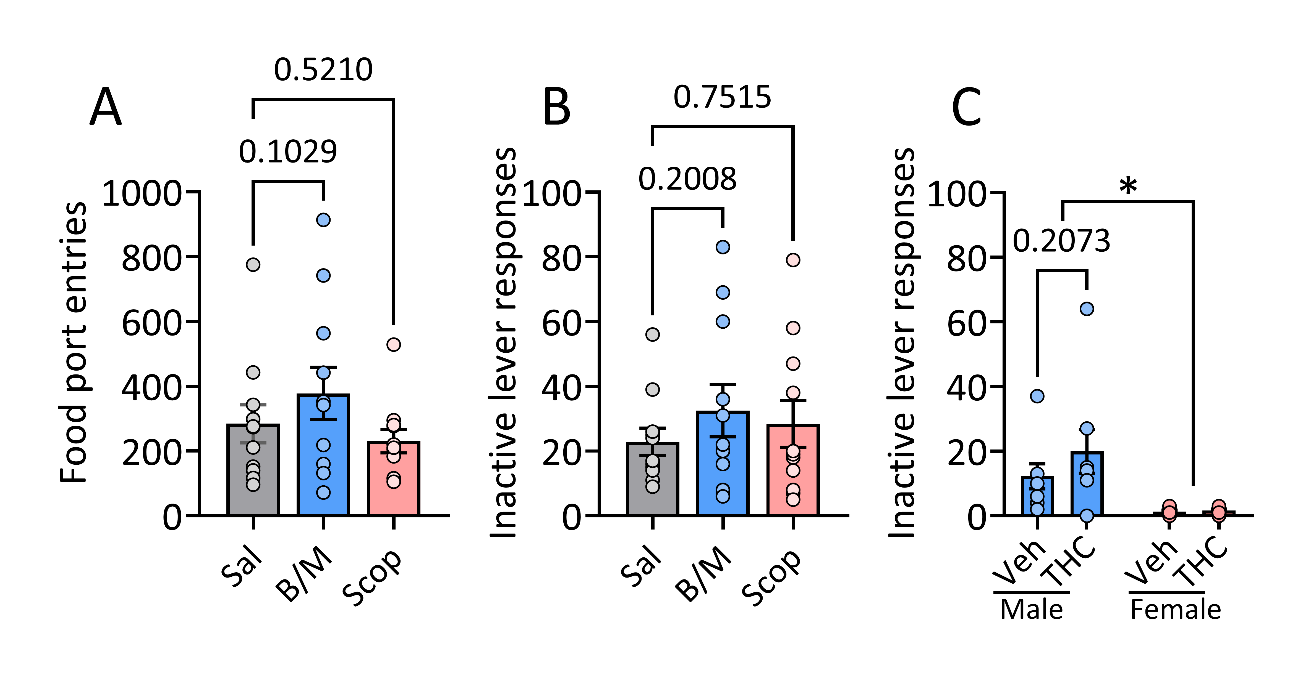


**Supplemental Figure 3**. **A-B.** Effects of intra-LHb infusion of Saline (Sal), baclofen/muscimol (B/M) or scopolamine (Scop) on Food port entries (**A**) and inactive lever responses (**B**) (n = 8 male and 3 female rats). Same group as shown in **Fig. 4**. **A.** Repeated measures 1-way ANOVA, F_1.667, 16.67_ = 3.709, p = 0.0532. **B.** Repeated measures 1-way ANOVA, F_1.243, 12.43_ = 0.5624, p = 0.5039. p-values for post-hoc comparisons using Dunnett’s test shown in each panel. **C.** inactive lever presses in male (n = 16) and female (n = 12 rats) after systemic injection of Δ^9^-THC (THC) or vehicle (Veh). * = significant main effect of sex using a mixed-effects ANOVA (F_1, 24_ = 10.26. p = 0.0038). The Sex x Drug interaction was not significant (F_1, 24_ = 0.66); nor was the effect of Δ^9^-THC on inactive lever presses in males significant (uncorrected Fisher's Least Significant Difference test, p = 0.2073.


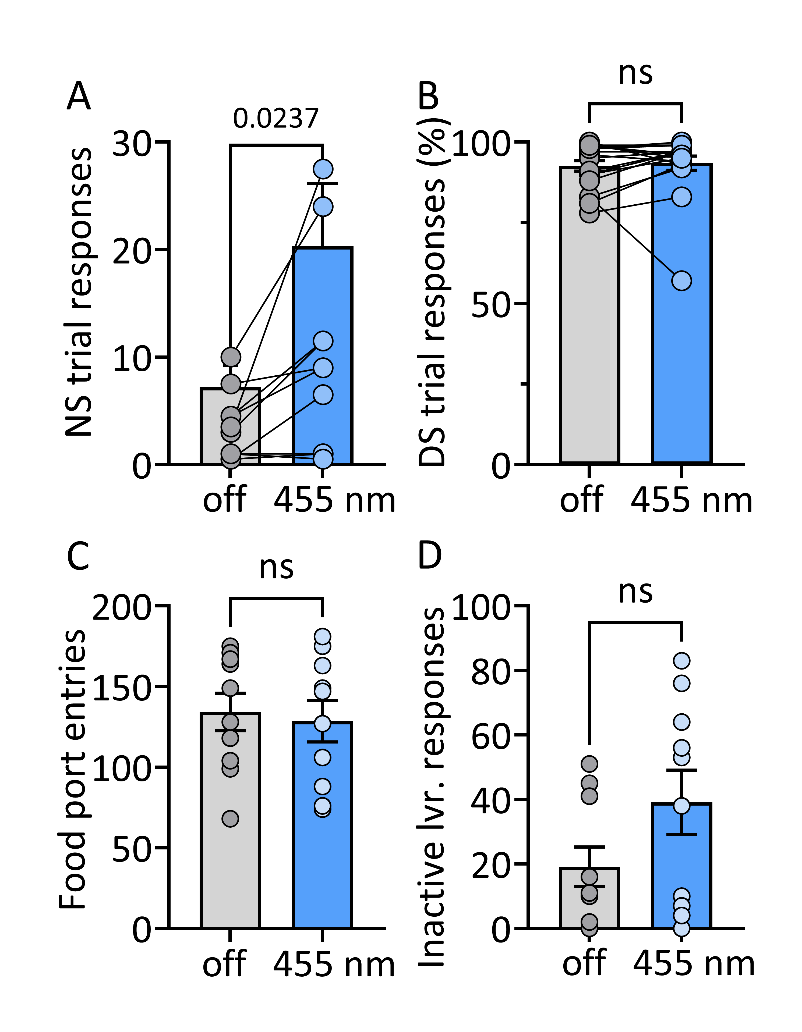
**Supplemental Figure 4.** Effects of photostimulation of ChR2 expressed on VP/NAcs axon terminals in the LHb on behavior in GADCre rats after injection of AAV-ChR2 into VP/NAcs. **A.** Same data as shown in **Fig. 5C**, here expressed as number of neutral stimulus (NS) trials in which responses on the active lever were observed in the absence and presence of ChR2 stimulation by 455 nm LED light. Activation of ChR2 significantly increased the number of responses during NS trials (n = 10 rats, t_9_ = 2.72, p = 0.0237, paired t-test). **B**. Photostimulation of ChR2 in the LHb did not significantly alter the percentage of discriminative stimulus (DS) trials in which responses were observed (n = 19 rats, t_18_ = 0.451, p = 0.657, paired t-test). **C**. Activation of ChR2 in VP/NAcs afferents in the LHb did not significantly alter the number of food port entries (n = 10 rats, t_9_ = 0.392, p = 0.703, paired t-test). **D**. Stimulation of ChR2 VP/NAcs afferents in LHb did not significantly affect the number of responses on the inactive lever (n = 10 rats, t_9_ = 1.93, p = 0.0857, paired t-test). Abbreviation: ns, non-significant.


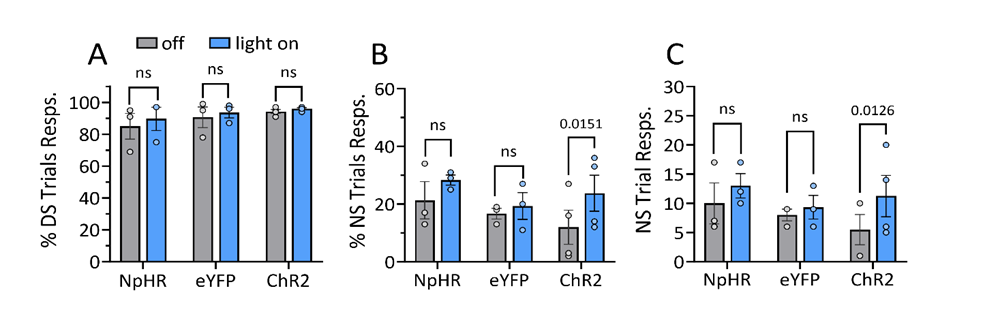
**Supplemental Figure 5.** Effects of optogenetic inhibition or activation of axon terminals in the LHb 8 weeks after infusion after injection of viral opsin constructs in the forebrain of wildtype rats. **A.** No effect of photoactivation (NpHR, 545 nm; ChR2, 455 nm, eYFP, 455 nm) of VP/NAcs-LHb axon terminals on operant responding during DS trials. **B.** Photostimulation of ChR2, virally expressed in VP/NAcs axon terminals projecting to LHb significantly increased responding during NS trials (RM-2 Way ANOVA, F_1,7_ = 14.63, p = 0.0065, p = 0.0151, Bonferroni post hoc comparison). In contrast, inhibition of these axons by photoactivation of NpHR had no effect on NS responding (p = 0.2306, Bonferroni). **C.** Same data shown in **B**, in which the number of responses during NS are shown for each condition (2-Way RM ANOVA, F_1, 7_ = 14.54, p = 0.067, p = 0.126, Bonferroni). The eYFP group represents control data obtained from rats injected with AAV-eYFP in the VP/NAcs with 455 nm LED stimulation in the LHb. These results are similar to those obtained with Cre-dependent expression of eYFP, NpHR, or Chr2 in GADcre rats (**Fig. 6C and 6D**, manuscript). Legend in panel **A** applies to all panels.

**References**

1 Ambroggi F, Ghazizadeh A, Nicola SM, Fields HL. Roles of nucleus accumbens core and shell in incentive-cue responding and behavioral inhibition. J Neurosci. 2011;31(18):6820-30.

2 Wolfe CIC, Hwang EK, Ijomor EC, Zapata A, Hoffman AF, Lupica CR. Muscarinic Acetylcholine M(2) Receptors Regulate Lateral Habenula Neuron Activity and Control Cocaine Seeking Behavior. J Neurosci. 2022;42(28):5552-63.

3 Marino M MM, Brogioli D. A new open source software for the calculation of the liquid junction potential between two solutions according to the stationary Nernst-Planck equation. arXiv preprint. 2014;1403.3640, 2014.

4 Paxinos G, Watson C. The Rat Brain in Stereotaxic Coordinates: Compact*.* Elsevier Science; 2008.
